# Retraction: MicroRNA-21 promotes TGF-β1-induced epithelial-mesenchymal transition in gastric cancer through up-regulating PTEN expression

**DOI:** 10.18632/oncotarget.28789

**Published:** 2025-12-31

**Authors:** Chang Li, Lei Song, Zhuo Zhang, Xiao-Xue Bai, Ming-Fu Cui, Lian-Jun Ma

**Affiliations:** ^1^Department of Gastrointestinal Surgery, China-Japan Union Hospital of Jilin University, Changchun, 130000, Jilin, P.R. China; ^2^Department of Respiratory Medicine, The First Hospital of Jilin University, Changchun, 130000, Jilin, P.R. China; ^3^Department of Orthopaedics, China-Japan Union Hospital of Jilin University, Changchun, 130000, Jilin, P.R. China; ^4^Department of Cadre Ward, The First Hospital of Jilin University, Changchun, 130000, Jilin, P.R. China; ^5^Department of Endoscopics, China-Japan Union Hospital of Jilin University, Changchun, 130000, Jilin, P.R. China

**This article has been retracted:** Oncotarget has completed its investigation of this paper. After a correction notice was published for this article in 2020, correcting an image in Figure 4D, several additional problems were identified. Specifically, Figures 5 and 6 have a lack of background noise, questioning the authenticity of the data. Also, the ‘Blank’ and ‘Negative Control’ images in the ‘48 hr’ row of Figure 11B are identical. In Figure 10B, a western blot duplicates one found in Figure 4J of [1], which has since been retracted. Figure 9B duplicates a western blot also shown in Figure 6G of [2], also retracted. Immunohistochemistry images in Figure 2C (P-Akt) and 2E (Akt) were reproduced in the unrelated papers [3, 4], respectively. And b-actin western blot in Figure 5C appeared in the later published article [5]. Requests for signed documentation to confirm the retraction were sent to all authors, but no signatures have been received. In light of these findings, the editorial decision was made to retract this paper.

Original article: Oncotarget. 2016; 7:66989–67003. 66989-67003. https://doi.org/10.18632/oncotarget.11888

## References

[1] Qiu Y , Cao X , Liu L , Cao X , Yuan Q , Li X , Cui Y , Xu C , Zou C , Ren K , Cao J . Modulation of MnSOD and FoxM1 Is Involved in Invasion and EMT Suppression by Isovitexin in Hepatocellular Carcinoma Cells. Cancer Manag Res. 2020; 12:5759–71. 10.2147/CMAR.S245283. . Retraction in: Cancer Manag Res. 2022; 14:1473–74. DOI: 10.2147/CMAR.S370617 PMID: 35469135 32765079 PMC7371559

[2] Liu F , Yuan Q , Cao X , Zhang J , Cao J , Zhang J , Xia L . Isovitexin Suppresses Stemness of Lung Cancer Stem-Like Cells through Blockage of MnSOD/CaMKII/AMPK Signaling and Glycolysis Inhibition. Biomed Res Int. 2021; 2021:9972057. 10.1155/2021/9972057. . Retraction in: Biomed Res Int. 2024; 2024:9802384. DOI: 10.1155/2024/9802384 PMID: 38230121 34195288 PMC8203360

[3] Han MY , Nie JW , Li YY , Zhu YZ , Wu G . Downregulation of NGAL is Required for the Inhibition of Proliferation and the Promotion of Apoptosis of Human Gastric Cancer MGC-803 Cells. Cell Physiol Biochem. 2018; 50:694–705. 10.1159/000494236. . Retraction in: Cell Physiol Biochem. 2021; 55:673. DOI: 10.33594/000000463 PMID: 34727447 30308516

[4] Ao R , Guan L , Wang Y , Wang JN . Silencing of COL1A2, COL6A3, and THBS2 inhibits gastric cancer cell proliferation, migration, and invasion while promoting apoptosis through the PI3k-Akt signaling pathway. J Cell Biochem. 2018; 119:4420–34. 10.1002/jcb.26524. . Retraction in: J Cell Biochem. 2021 (Suppl 1); 122:S102. DOI: 10.1002/jcb.30082 PMID: 34288045 29143985

[5] Wen Q , Xu C , Zhou J , Liu NM , Cui YH , Quan MF , Cao JG , Ren KQ . 8-bromo-7-methoxychrysin suppress stemness of SMMC-7721 cells induced by co-culture of liver cancer stem-like cells with hepatic stellate cells. BMC Cancer. 2019; 19:224. 10.1186/s12885-019-5419-5. . Retraction in: BMC Cancer. 2023; 23:1203. DOI: 10.1186/s12885-023-11698-1 PMID: 38062383 30866863 PMC6416872

